# Standard vs. Modified Antiplatelet Therapy Based on Thromboelastography With Platelet Mapping for Preventing Bleeding Events in Patients Undergoing Stent-Assisted Coil for a Ruptured Intracranial Aneurysm

**DOI:** 10.3389/fneur.2020.615829

**Published:** 2021-01-27

**Authors:** Yuanshu Li, Xiaodong Zhang, Zongduo Guo, Ji Zhu, Rui Xu, Zhaohui He, Xiaochuan Sun

**Affiliations:** Department of Neurosurgery, First Affiliated Hospital of Chongqing Medical University, Chongqing, China

**Keywords:** aneurysmal subarachnoid hemorrhage, thromboelastography, platelet function, stent-assisted coiling, antiplatelet therapy

## Abstract

**Background and Purpose:** Stent-assisted coiling (SAC) of intracranial aneurysms is usually treated with antiplatelet therapy to reduce the risk of postoperative ischemic events. However, using the same antiplatelet therapy for all patients may increase the risk of bleeding in patients with aneurysmal subarachnoid hemorrhage (aSAH). Thromboelastography-platelet mapping (TEG-PM) measures platelet function, which reflects the effect of antiplatelet drugs. This study aimed to evaluate the benefits of individualized antiplatelet regimens based on TEG-PM parameters for patients with aSAH who underwent SAC.

**Methods:** We retrospectively included patients with aSAH who treated with SAC during the period from June 2012 to December 2019. Patients were divided into two groups: patients whose antiplatelet therapy adjusted by TEG-PM parameters after surgery (adjustment group) and patients who were treated with standard dual antiplatelet therapy without TEG-PM test (control group). The occurrence of major/minor bleeding events, major/minor thromboembolic events, and favorable outcome (modified Rankin scale <3) were compared in both groups during hospitalization.

**Results:** Of 188 aSAH patients considered for this study, 145 met the criteria for inclusion and were included in the analysis (93 patients in the adjustment group and 52 patients in the control group). The risks of minor bleeding events (1.1 vs. 9.6%, *p* = 0.02) were significantly lower in patients in the adjustment group. However, there was no significant difference in the rate of major bleeding events at discharge between adjustment and control groups (*p* = 0.35). The rates of thromboembolic events and favorable outcome were similar in both groups (22.6 vs. 28.8%, *p* = 0.42, 95.7 vs. 96.2%, *p* = 1.00). Furthermore, the minor thromboembolic events rate was significantly lower in the patients treated with treatment plan C (*p* = 0.02 for treatment plan C vs. treatment A, *p* = 0.03 for treatment plan C vs. treatment plan B). However, there was no significant difference in the rate of other mentioned above complications and favorable outcomes among patients treated with different antiplatelet regimens.

**Conclusions:** Individualized antiplatelet therapy based on TEG-PM parameters might positively impact the bleeding risk of aSAH patients, without increasing the risk for clinically relevant thromboembolic events.

## Introduction

Stent treatment technology has emerged as a viable and preferable method for wide-necked, complex-shaped, and dissecting intracranial aneurysms (1). However, compared with coil embolization, stent-assisted coiling (SAC) of intracranial aneurysms has a higher incidence of perioperative complications and mortality (2, 3). Dual antiplatelet therapy (100 mg of aspirin and 75 mg of clopidogrel daily) has been wildly used to decrease the incidence of thromboembolic events in intracranial aneurysms treated with stents (4–6). Nevertheless, dual antiplatelet therapy also increased the likelihood of postoperative bleeding events (7). Especially for patients with aneurysmal subarachnoid hemorrhage (aSAH), once intracranial hemorrhage occurs, the prognosis will be severely deteriorated.

Thromboelastography-platelet mapping (TEG-PM) is a measure derived from thromboelastography (TEG) for detecting platelet function in the form of platelet inhibition rate, which can indirectly reflect the platelet aggregation function and can benefit to prevent and reduce thromboembolic events after embolization. Therefore, it is widely used in most hospitals around the world to evaluate the efficacy of dual antiplatelet agents before SAC for intracranial aneurysm (8). Current research have focused on the relationship between TEG-PM parameters and thromboembolic or bleeding events after SAC (9–12). Some studies (11–14) suggested that the parameter of TEG-PM could be identified as parameters for tailor-individualized antiplatelet treatment designed to reduce ischemic events and bleeding. However, there is little consensus regarding how to adjust the antiplatelet regiment according to TEG-PM parameters. The benefits of individualized antiplatelet therapy have not been well-investigated.

The purpose of this study is to explore whether the tailor reduction of the dose of antiplatelet drugs based on TEG-PM parameters can reduce the incidence of bleeding events after SAC without increasing the rate of thromboembolic events and whether it will affect the prognosis of patients.

## Methods

**Figure 1:**
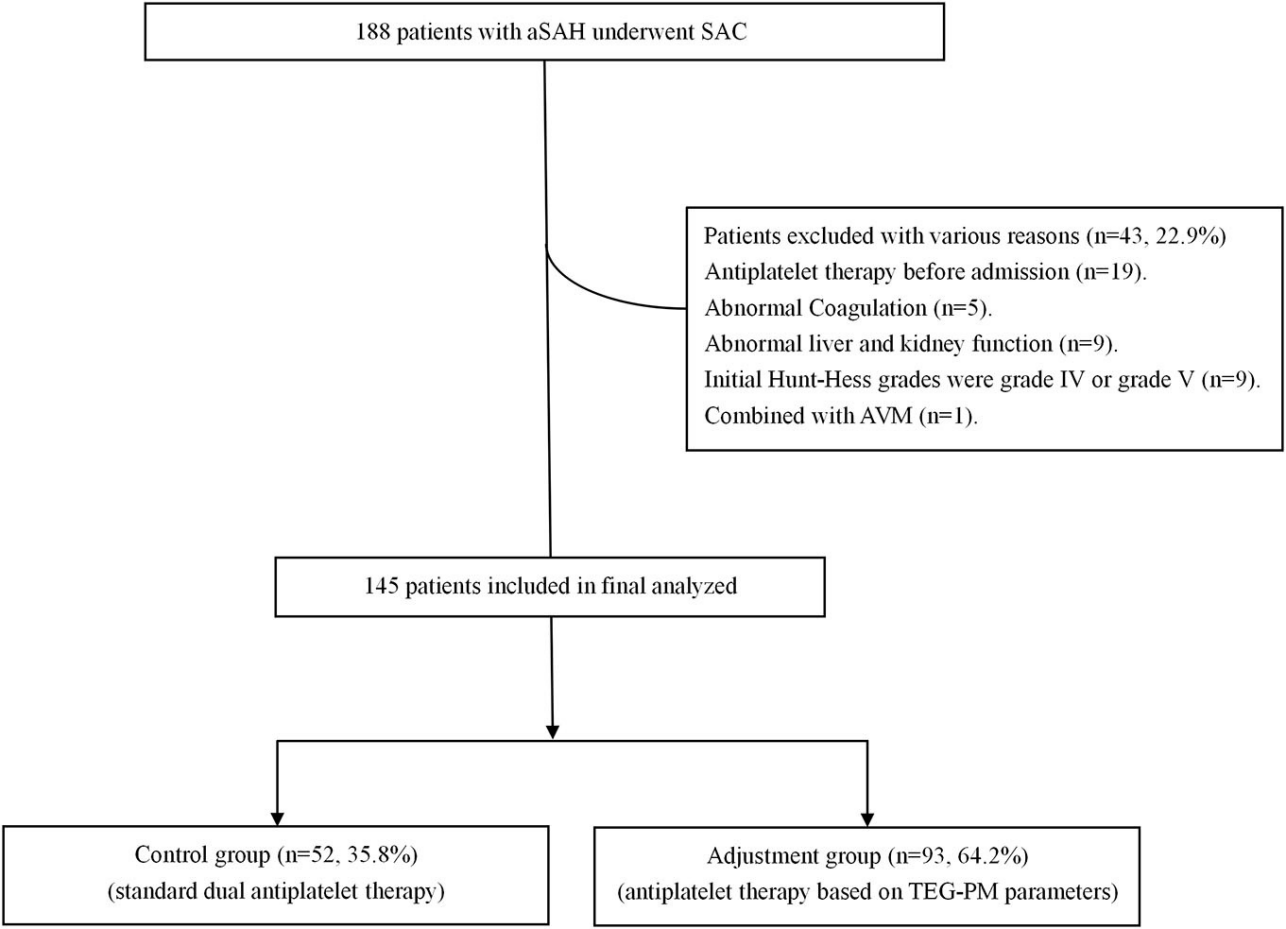
Abbreviated flowchart for patients included in this study based on the inclusion and exclusion criteria. aSAH, aneurysmal subarachnoid hemorrhage; SAC, stent-assisted coiling; AVM, arteriovenous malformations.

This study was a single-institution retrospective study conducted at The First Affiliated Hospital of Chongqing Medical University. The STROBE statement on cohort studies guided study design and manuscript organization. We retrospectively recruited patients with aSAH who underwent SAC between June 2012 and December 2019. Patients who were treated with an antiplatelet regimen based on TEG-PM parameters due to SAC treatment were included into an “adjustment” group. A “control” group consisting of aSAH patients who underwent SAC without TEG-PM test during the period from June 2012 to January 2015 was added to the final analysis (Figure 1). A further subgroup analysis was performed within the adjustment group, comparing patients with different antiplatelet therapy. The baseline demographic information, including age, sex, Hunt-Hess grades, modified Rankin scale (mRS) score, procedure time, cerebrovascular risk factors (smoking and drinking history, diabetes, hypertension), aneurysm characteristics (location, size), multiple aneurysms, stent types, and platelet count were recorded. The initial clinical condition of the patients was graded by Hunt-Hess grade, and we only included 1–3 grades into the analysis. This study was approved by the institutional review board of The Frist Affiliated Hospital of Chongqing Medical University, in accordance with the Declaration of Helsinki. Due to the retrospective nature of the study, informed consent was waived.

### Inclusion/Exclusion Criteria

Inclusion criteria were as follows: (1) Patients (ages ≥18 years) with aSAH were treated with SAC were included; (2) have complete perioperative-related imaging and laboratory data; and (3) Initial Hunt-Hess grades were grades I–III. Exclusion criteria included the following: (1) treatment with anticoagulants, thrombolytic agents, and other antiplatelet drugs before admission; (2) severe cardiovascular disease or cerebral ischemic stroke; (3) severe hepatic or renal dysfunction, malignant disease, chronic inflammatory disease, significant coagulopathy, or an infectious condition at study entry; (4) combined with other cerebral diseases; and (5) recurrent aneurysm which is treated with coils only or stent.

### Management Protocol

A standardized protocol was strictly followed in all cases. All cases were treated according to the standardized aSAH treatment protocol (15) consisting of absolute bed rest until endovascular treatment, strict blood pressure control, intravenous administration of hemostatic agents and nimodipine, and regular assessment of clinical status. All patients were treated with 300 mg of aspirin and 300 mg of clopidogrel at least 2 h before the procedure. The endovascular procedure was performed under general anesthesia, and a bolus of heparin was administered using 3,000 IU, then 1,000 IU every hour. The Enterprise (Codman Neurovascular), Solitaire AB neurovascular remodeling device (eV3, Inc.), LVIS (MicroVention Terumo, Tustin, California, USA), and LEO stents (Balt, Montmorency, France) stents were used to treat aneurysms. All patients discontinued heparin on day 3 postoperatively, and patients in the adjustment group would be treated with the antiplatelet regimen based on TEG-PM parameters. Patients in the control group would be treated with standard dual antiplatelet therapy (aspirin 100 mg and clopidogrel 75 mg).

### Laboratory Examination

Patients in the adjustment group were drawn blood samples for TEG and TEG-PM testing on the 3rd day after surgery draws blood. Blood samples (3–4 ml) were drawn from a single clean puncture of a forearm vein and collected in a 4-ml test tube (Becton-Dickinson) containing lithium heparin.

The platelet inhibition was tested using a TEG-PM analyzer (Haemoscope, Model 5000). Adenosine diphosphate (ADP) agonists and arachidonic acid (AA) were added to measure the platelet inhibition of the P2Y12 receptor and cyclooxygenase pathways. Inhibition rate (%) is equal to (AA- or ADP-induced clot strength-fibrin clot strength)/(thrombin-induced clot strength-fibrin clot strength) × 100% (16). The percentage of platelet inhibition in response to ADP (ADPi) and MA-ADP (ADP-induced clot strength) were used to measure the response to clopidogrel. The percentage of platelet inhibition in response to AA (AAi) was used to measure aspirin's response.

The following TEG parameters were recorded: the time from the start of the sample until amplitude of clot formation reaches 2 mm (*R*, min); the time elapsed from *R* until 20-mm amplitude is achieved (*K*, min); the time to reach maximum speed of initial clot formation (α angle, deg); maximum clot strength (MA, mm); percent of clot lysis 30 min after MA (EPL); and percent of amplitude decay 30 min after MA (LY30).

### Antiplatelet Regimen

**Figure 2:**
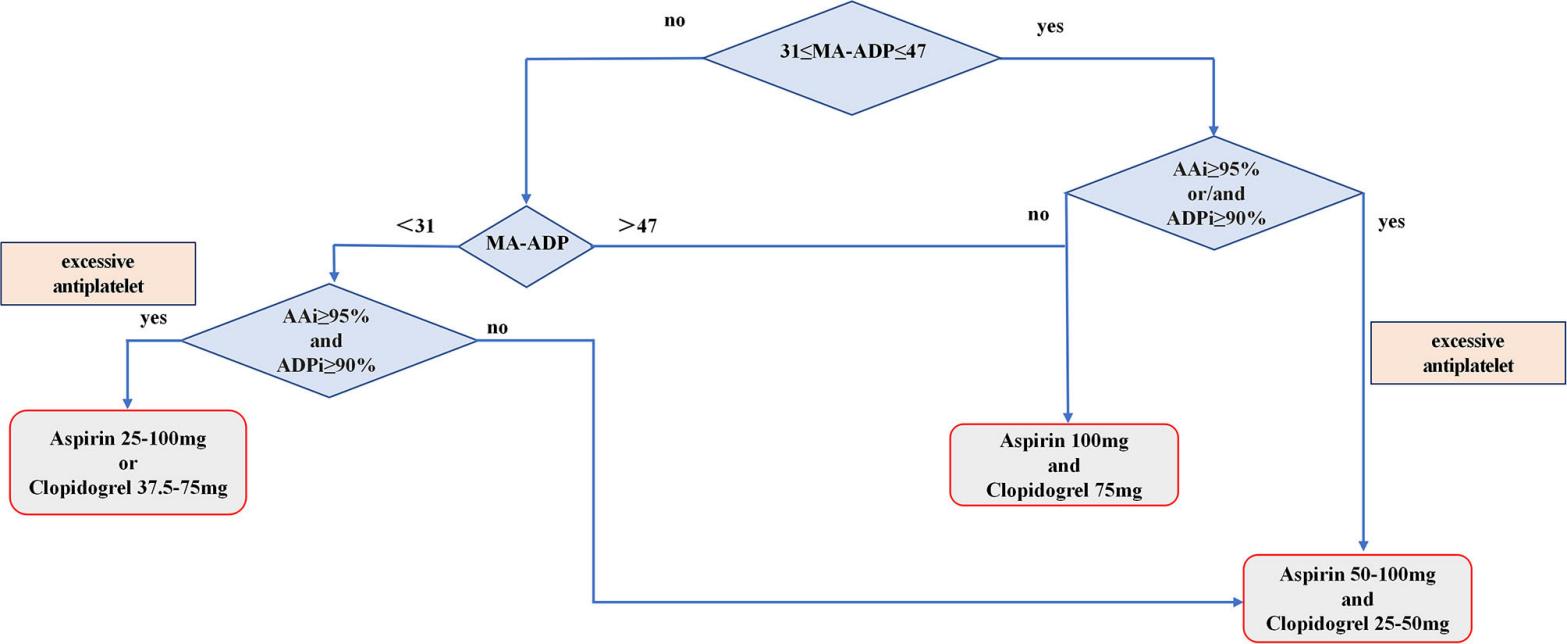
Flowchart showing the process of patients receive individualize antiplatelet therapy based on TEG-PM parameters.

Patients in the control group were treated with standard dual antiplatelet after surgery due to the TEG-PM test has not been applied in our department between June 2012 and January 2015. Patients in the adjustment group were treated with an antiplatelet regimen adjusted according to TEG-PM parameters on the 3rd day after surgery. The specific process of individualized antiplatelet therapy is shown in Figure 2 which includes three antiplatelet treatment options, as follows: (1) When MA-ADP <31, if AAi ≥ 95% and ADPi ≥ 90%, choose treatment plan A (aspirin, 25–100 mg or clopidogrel, 37.5–75 mg), otherwise use treatment plan C (aspirin, 50–100 mg and clopidogrel 25–50 mg); (2) When 31 ≤ MA-ADP ≤ 47, if AAi ≥ 95%, and/or ADPi ≥ 90%, use treatment plan C (aspirin, 50–100 mg and clopidogrel 25–50 mg); otherwise, use treatment plan B (aspirin, 100 mg and clopidogrel, 75 mg); (3) When MA-ADP > 47, use treatment plan B (aspirin, 100 mg and clopidogrel, 75 mg); and (4) If the patient's treatment plan is A or C, the specific antiplatelet drug dose is based on the value of AAi, ADPi, and MA-ADP values. The higher the value of AAi and ADPi, the lower the dose of antiplatelet drugs. The lower the value of MA-ADP, the lower the dose of antiplatelet drugs. During hospitalization, if patients have symptoms of bleeding or ischemia, the dose of antiplatelet drugs should be increased or decreased according to the signs. All patients in the adjustment group adjusted antiplatelet therapy based on TEG-PM parameters again when they were discharged from the hospital and 6 weeks after discharge. If the patient has no symptoms of bleeding or ischemia, the adjusted antiplatelet drug regimen will be maintained until 6 months. The total duration of antiplatelet therapy for patients in this study was 6 months.

### Complications and Outcomes

Trained neurosurgeon reviewed all follow-up CT/MRI scans during the period of antiplatelet therapy (4 days after surgery to the time of discharge) for any clinical information about the occurrence of cerebral infarctions and new intracranial bleedings. Minor bleeding was defined as any extracranial bleeding (gastrointestinal hemorrhage, ecchymosis, epistaxis, and hematuria) documented during antiplatelet therapy that does not cause clinical deterioration. Major bleeding was defined as any new hemorrhage that led to clinical deterioration. Accordingly, a minor thromboembolic event was defined as new asymptomatic cerebral infarction in the stent vessel area, which was diagnosed by diffusion-weighted imaging (DWI). Major thromboembolic events included newly developed transient ischemic attack (TIA) or symptomatic ischemic infarctions. We followed up with patients in the adjustment group via hospital medical records or telephone interviews in July 2020, and the median follow-up time was 11 (7, 19) months. Functional outcome was evaluated at discharge and at least 6 months after discharge. Due to the large time span of patients in the control group and the adjustment group, this study only followed up patients in the adjustment group. Favorable functional outcome was defined as mRS <3.

### Statistical Analysis

Data are presented as mean SD or expressed in terms of frequency and percentage. Categorical variables were analyzed using the Chi-square test or, if applicable, with the Fisher exact test. Continuous variables were analyzed using the Student's *t*-test for normally distributed and the Mann–Whitney *U*-test for non-normally distributed data. *p* < 0.05 was considered statistically significant. The statistical analysis was performed by SPSS 23.0 software (SPSS Inc., Chicago, Illinois, USA).

## Results

### Population

Of 188 aSAH patients enrolled for this study, 145 endovascularly treated individuals (77.5%) met the inclusion criteria and were included in the final analysis (see Figure 1). The mean age of these patients at admission was 54 ± 11 years. The majority (99, 68.3%) were females. One hundred and seventy-six stents were placed in patients. Table 1 shows the baseline characteristics of both adjustment and control groups. There are no statistically significant differences between the groups for age, sex, procedure time, Hunt-Hess grade, medical history, aneurysm location and size, multiple aneurysms, platelet count, and stent types (see Table 1).

**Table 1:** Baseline characteristics of the patients in the adjustment and control groups.

**Variable (No. %)**	**Adjustment (*n* = 93)**	**Control (*n* = 52)**	***p*-values**
Age (mean ±*SD*, years)	53 ± 11	54 ± 10	0.77
Female sex	61 (65.6)	38 (73.1)	0.45
**Hunt-Hess grade**			0.24
Grade I	15 (16.1)	6 (11.5)	
Grade II	74 (79.6)	40 (76.9)	
Grade III	4 (4.3)	6 (11.5)	
Procedure time (IQR, min)	120 (95–140)	120 (102–160)	0.87
**Medical history**						
Hypertension	41 (44.1)	23 (44.2)	1.00
Diabetes mellitus	9 (9.7)	3 (5.8)	0.53
Smoking	24 (25.8)	14 (26.9)	1.00
Drinking	15 (16.1)	8 (15.4)	1.00
Anterior circulation aneurysm	81 (87.1)	46 (88.5)	1.00
Aneurysm size ≥10 mm	14 (15.1)	6 (11.5)	0.62
Multiple aneurysms	24 (25.8)	7 (13.5)	0.09
**Stent types**			0.14
Enterprise	52 (55.9)	24 (46.2)	
LVIS	8 (8.6)	4 (7.7)	
Solitaire AB	30 (32.3)	17 (32.7)	
LEO	3 (3.2)	7 (13.5)	
PLT^a^ [k/μl (Med, IQR)]	196 (165–236)	216 (169–277)	0.08

*SD, standard deviation; PLT, platelet; Med, median; IQR, interquartile range*.

a *Postprocedure*.

There was also no statistical difference in baseline characteristics between patients with different antiplatelet treatment plans in the adjustment group (see Table 2). Six (6.5%) patients were lost to follow-up in the adjustment group. The proportion of different antiplatelet treatment plan in the adjustment group is as follows: treatment plan A (49, 52.7%, aspirin, 25–100 mg or clopidogrel, 37.5–75 mg); treatment plan B (28, 30.1%, aspirin, 100 mg and clopidogrel, 75 mg); and treatment plan C (16, 17.2%, aspirin 50–100 mg and clopidogrel, 25–50 mg).

**Table 2:** Baseline characteristics of the patients who underwent SAC with individualized antiplatelet therapy.

**Variable (No. %)**	**Treatment plan A**	**Treatment plan B**	**Treatment plan C**	***p*****-values**		
	**ASA, 25–100 mg or CLOP, 37.5–75 mg**	**ASA, 100 mg and CLOP, 75 mg**	**ASA, 50–100 mg and CLOP, 25–50 mg**	**A vs. B**	**A vs. C**	**C vs. B**
Number of patients	49 (52.7)	28 (30.1)	16 (17.2)			
Age (mean ±*SD*, years)	52 ± 11	57 ± 12	51 ± 11	0.07	0.68	0.10
Female sex	15 (30.6)	8 (28.6)	9 (56.3)	1.00	0.08	0.10
**Hunt-Hess grade**				0.89	1.00	1.00
Grade I	9 (18.4)	4 (14.3)	2 (12.5)			
Grade II	38 (77.6)	23 (82.1)	13 (81.3)			
Grade III	2 (4.1)	1 (3.6)	1 (6.3)			
Procedure time (IQR, min)	125 (100-140)	120 (91-143)	120 (96-157)	0.45	0.51	0.83
**Medical history**						
Hypertension	19 (38.8)	17 (60.7)	5 (31.3)	0.09	0.73	0.11
Diabetes mellitus	2 (4.1)	5 (17.9)	2 (12.5)	0.09	0.25	1.00
Smoking	11 (22.4)	6 (21.4)	7 (43.8)	1.00	0.11	0.17
Drinking	7 (14.3)	4 (14.3)	4 (25.0)	1.00	0.44	0.43
Anterior circulation aneurysm	44 (89.8)	22 (78.6)	15 (93.8)	0.19	1.00	0.39
Aneurysm size ≥10 mm	10 (20.4)	4 (14.3)	0	0.55	0.05	0.28
Multiple aneurysms	9 (18.4)	8 (28.6)	7 (43.8)	0.39	0.05	0.34
**Stent types**				0.81	0.43	0.13
Enterprise	26 (53.1)	18 (64.3)	8 (50.0)			
LVIS	5 (10.2)	3 (10.7)	0			
Solitaire AB	16 (32.7)	6 (21.4)	8 (50.0)			
LEO	2 (4.1)	1 (3.9)	0			
**Post-procedure laboratory test**						
PLT [k/μl (Med, IQR)]	196 (164–232)	193 (168–230)	205 (159–269)	0.88	0.72	0.75
*R* [min (Med, IQR)]	5.6 (4.7–6.2)	5.6 (5.0–6.7)	4.8 (4.2–5.7)	0.88	0.21	0.34
*K* [min (Med, IQR)]	1.3 (1.2–1.6)	1.3 (1.2–1.5)	1.2 (1.0–1.3)	0.91	0.17	0.39
α-Angle [deg (Med, IQR)]	70.6 (67.0–72.9)	69.9 (67.7–71.9)	73.2 (67.9–74.5)	0.53	0.13	0.11
MA [mm (Med, IQR)]	67.8 (64.9–71.0)	67.6 (63.4–70.8)	68.7 (64.4–71.2)	0.98	0.72	0.34
EPL [% (Med, IQR)]	0.9 (0.2–1.8)	1.0 (0.1–3.3)	0.8 (0.1–3.9)	0.88	0.82	0.75
LY30 [% (Med, IQR)]	0.8 (0.1–1.7)	0.5 (0.1–3.0)	0.7 (0.1–2.2)	0.55	0.94	0.58

*SAC, stent-assisted coiling; SD, standard deviation; vs., versus; PLT, platelet; Med, median; IQR, interquartile range; R, reaction time to clot formation; K, clotting time until 20-mm amplitude is achieved; α-Angle, time to reach maximum speed of initial clot formation; MA, maximum amplitude; EPL, percent of clot lysis 30 min after MA; LY30, percent of amplitude decay 30 min after MA*.

### Antiplatelet Therapy and Bleeding Events

The adjustment group had a significantly decreased incidence of minor bleeding events compared with the control group (1.1 vs. 9.6%, *p* = 0.02). But there was no significant difference in the rate of major bleeding events at discharge between adjustment and control groups (*p* = 0.35, see Table 3). One patient in the adjustment group experienced gastrointestinal bleeding 11 days after surgery. After adjusting the antiplatelet regimen to aspirin 100 mg, the patient did not show any bleeding symptoms during hospitalization. There were six bleeding patients in the control group, including intracranial hemorrhage (1, 1.9%), gastrointestinal bleeding (1, 1.9%), hematuria (2, 3.8%), and ecchymosis (2, 3.8%). After symptomatic treatment, the bleeding symptoms disappeared and the patient with intracranial hemorrhage did not develop neurological deficits. Telephone follow-up found that four patients in the adjustment group had intracranial hemorrhage due to improper blood pressure control, all of which occurred after being transferred to a local hospital for treatment. The rate of minor/major bleeding events at discharge and major bleeding events during follow-up was compared among the patients with a different treatment plan within the adjustment group, and we find no significant difference among them (see Table 4).

**Table 3:** Complications and favorable outcomes in patients between adjustment and control groups.

**Variable (No. %)**	**Adjustment (*n* = 93)**	**Control (*n* = 52)**	***p*-values**
**Bleeding events**			
Minor at discharge	1 (1.1)	5 (9.6)	0.02
Major at discharge	0	1 (1.9)	0.35
**Thromboembolic events**			
Minor at discharge	20 (21.5)	15 (28.8)	0.41
Major at discharge	1 (1.1)	0	1.00
**Favorable outcomes**			
mRS <3 at discharge	89 (95.7)	50 (96.2)	1.00

*mRS, modified Rankin scale*.

**Table 4:** Complications and favorable outcomes in patients who underwent SAC with individualized antiplatelet therapy.

**Variable (No. %)**	**Treatment plan A**	**Treatment plan B**	**Treatment plan C**	***P*****-values**		
	**ASA, 25–100 mg or CLOP, 37.5–75 mg**	**ASA, 100 mg and CLOP, 75 mg**	**ASA 50–100 mg and CLOP, 25–50 mg**	**A vs. B**	**A vs. C**	**C vs. B**
Number of patients	49 (52.7)	28 (30.1)	16 (17.2)	
**Bleeding events**						
Minor at discharge	0	1 (3.5)	0	0.36	-	1.00
Major at discharge	0	0	0	-	-	-
Major during follow-up	1 (2.0)	2 (7.1)	1 (6.3)	0.55	0.43	1.00
**Thromboembolic events**						
Minor at discharge	13 (26.5)	7 (25.0)	0	1.00	0.02	0.03
Major at discharge	1 (2.0)	0	0	1.00	1.00	-
Major during follow-up	0	0	0	-	-	-
**Favorable outcomes**						
mRS <3 at discharge	48 (2.0)	25 (89.3)	16 (100)	0.13	1.00	0.29
mRS <3 at least 6 months^a^	42 (97.7)	26 (92.9)	16 (100)	0.55	1.00	0.52

*SAC, stent-assisted coiling; vs, versus; mRS, modified Rankin scale; ASA, aspirin; CLOP, clopidogrel*.

a *87 patients were followed up at least 6 months after discharge*.

### Antiplatelet Therapy and Thromboembolic Events

There was no significant difference in the rate of minor/major thromboembolic events at discharge between adjustment and control groups (*p* = 0.41 for minor, *p* = 1.00 for major, see Table 3). One patient in the adjustment group developed TIA during antiplatelet therapy. After adjusting the patient's antiplatelet regimen from aspirin 100 mg to aspirin 100 mg and clopidogrel 75 mg, the ischemic symptoms did not recur. No patients in the control group developed major thromboembolic events. The rate of minor/major thromboembolic events at discharge and major thromboembolic events during follow-up was compared among the patients with a different treatment plan within the adjustment group, and we find that the minor thromboembolic events rate was significantly lower in the patients treated with treatment plan C (*p* = 0.02 for treatment plan C vs. treatment A, *p* = 0.03 for treatment plan C vs. treatment plan B, see Table 4).

### Antiplatelet Therapy and Outcome

The rate of favorable outcomes at discharge in the adjustment group was not statistically different from that in the control group (*p* = 1.00 at discharge, see Table 3). Furthermore, we find that there is no significant difference in the rate of favorable outcomes at discharge or least 6 months after discharge among patients with a different antiplatelet treatment plan within the adjustment group (*p* = 0.13, *p* = 0.55 for treatment plan A vs. treatment B, *p* = 1.00, *p* = 1.00 for treatment plan A vs. treatment C, *p* = 0.29, *p* = 0.52 for treatment plan C vs. treatment B, see Table 4).

## Discussion

In this retrospective cohort study, we analyzed the benefits and risks associated with the tailor reduction of the dose of antiplatelet drugs based on TEG-PM parameters in patients with aSAH after SAC. In addition, we find that the individualized antiplatelet therapy based on TEG-PM parameters could reduce the bleeding risks of patients with aSAH after SAC without increasing the rates of thromboembolic events and unfavorable outcomes.

### TEG-PM Parameters and Antiplatelet Therapy

Current studies show that ADPi, AAi, and MA-ADP values can predict the risk of ischemic events after SAC of intracranial aneurysms. Lower ADPi, AAi, and higher MA-ADP values and thromboembolic events after SAC of intracranial aneurysms are related. On the other hand, a higher ADPi value can predict bleeding events after SAC of intracranial aneurysms (9, 12, 13). However, the definition of TEG-PM parameter safety ranges is different in different studies. The results of the study by Ge et al. showed that the safe range of ADPi in patients after SAC of intracranial aneurysms is 29.45–55.4%, and the safe range of MA-ADP is <46.15, and they suggest that ADPi < 29.45% or MA-ADP > 46.15 need aggressive antiplatelet therapy (10). According to Wang et al. study, MA-ADP > 49.95 mm is the best cut-off value for predicting ischemic events after SAC of intracranial aneurysms (9). Moreover, many studies on percutaneous coronary intervention (PCI) was defined AAl < 50% and ADPi < 30% as resistance to aspirin and clopidogrel, respectively, and showed that resistance to aspirin and clopidogrel is associated with the high thromboembolic risk of the patients after PCI (17–20). However, thromboembolic events are undoubtedly multifactorial, and increasing the dose of antiplatelet drugs for patients with aSAH due to the “resistance” is more likely to increase the risk of bleeding after endovascular treatment. In this study, given that there is no consensus on the definition of the safety range of TEG-PM parameters, and the definition of resistance is affected by multiple factors, we tailored to reduce the dose of antiplatelet drugs for patients with excessive antiplatelet indicated by the TEG-PM parameters.

### Individualized Antiplatelet Therapy Impact on Complications and Outcome

Previous large-scale cardiovascular prospective clinical studies (ANTARCTIC, ARCTIC, GRAVITAS) (21–23) have shown that individualized antiplatelet therapy which was based on the results of VerifyNow platelet function testing cannot reduce cardiovascular mortality and long-term ischemia in patients after PCI. However, given the characteristics of shorter use time of antiplatelet drugs in neuro-interventional therapy and a higher risk of postoperative bleeding in patients with ruptured aneurysms, TEG-PM parameters are used for adjusting antiplatelet drugs to reduce complications after SAC of intracranial aneurysms deserves further study. Some studies suggested that patients who underwent SAC of intracranial aneurysms have been associated with perioperative ICH, and some studies (24–26) indicate that P2Y12 receptor over-inhibition may play a role. The Japanese Registry of Neuroendovascular Therapy (JR-NET) which was a nationwide survey from January 2005 to December 2009 found that there was a significant decreased rate of ischemic complications (4.2–2.1%) but a significantly increased rate of intracranial hemorrhagic complications (2.1–5.3%), as well as a significantly increased rate in death or severe disability (1.5–2.1%) for those patients who receive endovascular treatment with antiplatelet therapy (27). Congruently, Darkwah et al. also reported that there was an increased risk for major bleeding events in aneurysmal subarachnoid hemorrhage patients with dual antiplatelet therapy, as compared with aspirin monotherapy (7). In our study, the adjustment group's bleeding rate was significantly lower than that of the control group which consistent with the findings of Darkwah et al. Only one patient in the adjustment group had a bleeding event (gastrointestinal bleeding) on the 11th day after surgery whose AAi was 12.4%, ADPi was 69.5%, and MA-ADP value was 35.4. Although this TEG-PM parameter does not indicate excessive antiplatelet, bleeding events still occurred. The cause of bleeding events may be that TEG parameters can only represent the patient's platelet function during a period, suggesting that TEG-PM needs to be reviewed in time to assess changes in platelet function. McTaggart et al. (28) also use TEG-PM parameters to tailor dual-antiplatelet therapy in patients undergoing treatment of intracranial aneurysms with the Pipeline embolization device and followed up for 1 year. They found that none of the 31 patients had bleeding events. However, since the study by McTaggart et al. is a descriptive observational study and the sample size is small, further research is needed to use TEG-PM as a tool to identify the subset of patients who are at highest risk for bleeding events.

On the other hand, increasing the dose of antiplatelet drugs to reduce the risk of thromboembolic in patients with SAC of intracranial aneurysms needs to be further studied. Although McTaggart et al. (28) changed the clopidogrel hypo-responders' antiplatelet regimen, the thromboembolic risk for patients who underwent pipeline embolization did not eliminate. In their study, clopidogrel hypo-responders were either reloaded with clopidogrel 600 mg before the procedure or changed over to prasugrel before the procedure or the next day using the IIb/IIIa antagonist Integrilin as an effective antiplatelet bridge. Our study showed that despite the reduced dose of antiplatelet drugs, the incidence of minor/major thromboembolic events in the adjustment group was not higher than in the control group. Furthermore, the incidence of minor ischemic events in patients with treatment plan C was also significantly lower than that of patients with treatment plan A or B. The results mentioned above suggest that the preventive effect of increasing antiplatelet drugs on minor/major thromboembolic events needs further study. The results of Price et al. (29) showed that increasing the dose of antiplatelet drugs in patients with platelet hyperresponsiveness after PCI failed to reduce postoperative cardiogenic mortality and the incidence of non-fatal myocardial infarction or stent thrombosis which also support this view.

Besides, the incidence of TIA in the adjustment group of this study (1.1%; 1/93) and the incidence of minor thromboembolic events (21.5%; 20/93) were lower than the reported incidence in the literature [1.9% (30) for TIA, 43% (31) for minor thromboembolic events], which also showed that targeting to reduce the doses of antiplatelet drugs in patients with high bleeding risk did not lead to an increase in the incidence of ischemic events.

Finally, there is no significant difference between adjustment and control groups with regard to favorable outcomes at discharge. This finding is in line with several previous publications reporting thromboembolic events are not an important risk factor for poor prognosis in aSAH patients treated with SAC of intracranial aneurysms (32, 33). Therefore, the primary goal of individualized antiplatelet therapy based on TEG-PM parameters after SAC of intracranial aneurysms should be to identify people at high risk of bleeding and reduce the dose of antiplatelet drugs to reduce the risk of bleeding.

## Conclusion

Individualized antiplatelet therapy based on TEG-PM parameters might positively impact the bleeding risk of aSAH patients, without increasing the risk for clinically relevant thromboembolic events. However, our data underline the need for a prospective randomized trial for a definite safety range of TEG-PM parameters in individuals with aSAH.

## Limitations

There are some limitations to our study. First, we included only the patients whose initial Hunt-Hess grades were grades I–III. Therefore, our results and conclusions on the benefits of individualized antiplatelet therapy based on TEG-PM parameters after SAC of intracranial aneurysms cannot be generalized on aSAH patients whose initial Hunt-Hess grades were grades IV–V. Second, the dynamic of ADPi, AAi, and MA-ADP values during follow-up was lacking due to our retrospective design. Moreover, 6.5% of patients in the adjustment group were lost to follow-up, and it cannot be excluded that some of them could have been dead for cerebrovascular events. Last, this was a retrospective study with a small sample size, so further studies are required to support our findings.

## Data Availability Statement

The raw data supporting the conclusions of this article will be made available by the authors, without undue reservation.

## Ethics Statement

The studies involving human participants were reviewed and approved by the institutional review board of The First Affiliated Hospital of Chongqing Medical University. The ethics committee waived the requirement of written informed consent for participation.

## Author Contributions

YL: conception, collection of data, interpretation of data, and drafting the work. XZ, ZG, JZ, RX, and ZH: collection of data and interpretation of data. XS: conception and revising the work critically for important intellectual content. All authors contributed to the article and approved the submitted version.

## Conflict of Interest

The authors declare that the research was conducted in the absence of any commercial or financial relationships that could be construed as a potential conflict of interest.
